# Invited commentary: The Covid-19 pandemic in the United States

**DOI:** 10.1186/s12939-020-01354-6

**Published:** 2021-01-04

**Authors:** Neil A. (Tony) Holtzman

**Affiliations:** Johns Hopkins School of Medicine (Emeritus), Baltimore, MD USA

## Abstract

Despite being the wealthiest and one of the most technologically advanced countries in the world, the United States has the greatest number of Covid-19 cases and deaths. What accounts for this failure? The dismantling of the country’s public health infrastructure has crippled contact tracing and exacerbated inequality as a disproportionate number of poor people and people of color have fallen ill with Covid-19. Inadequate regulation of the private for-profit sector has adversely affected the efficiency and quality of testing for the virus, and the prescription of costly drugs whose benefit and safety in treating infected patients have not been established. More stringent regulation of the commercial sector has led to the development of efficacious vaccines in a remarkably short time. Still, questions remain about the vaccines’ effectiveness in the real world, and their safety.

In December 2020, the United States had the most cases of, and deaths from, Covid-19 among the 220 countries that report data; the fourth most cases per capita; and the eighth most deaths per capita [1].^1^ How could the wealthiest and one of the most technologically advanced countries in the world fare so poorly?

President Donald Trump’s pursuit of reelection shaped his approach to the pandemic: he willfully and repeatedly denied the severity of the pandemic, and disparaged face masks and safe distancing. Had not many state, city, and county officials insisted on masks and restricted public events and business openings, the toll would have been higher. Believing that restoring jobs would gain him votes, Trump ignored public health authorities’ warning that reopening society before containing the pandemic would cause a resurgence of Covid-19. With some reopening, the unemployment rate did fall from a high of 14.7% in April 2020 to 6.7% in November but close to 11 million people remained unemployed [2], the surges have materialized [1], and Trump did not win reelection.

Not surprisingly, Trump’s appointments—the Commissioner of the Food and Drug Administration (FDA), the Director of the Centers for Disease Control and Prevention (CDC), his cabinet—and the Republican U.S. Senate majority followed his lead. During Trump’s presidency, dismantling of the public health infrastructure and deregulation of the private sector both accelerated. The President and his administration undermined diagnostic testing and contact-tracing, and exacerbated inequality. Vaccine development has fared better. I explore these issues in this commentary.

## Dismantling the public health response

Over the past 15 years, federal funding for public health preparedness has been reduced by 28% [3]. Between 2008 and 2017, more than 55,000 positions were eliminated from local health departments. Before Covid-19 reached the U.S., President Trump’s proposed 2021 budget for the Centers for Disease Control and Prevention (CDC) was 10% less than the previous year, including a cut of $85.3 million in its program for emerging infectious diseases [4]. In 2018, the National Security Council’s Global Health Security and Biodefense unit, established in 2015 by the Obama Administration after the Ebola outbreak in 2014, was largely disbanded [5].

Judges appointed by a Republican majority in the Senate are also upending a historical tradition of upholding state laws that protected the public’s health, including inspection, quarantine, and vaccination [6].

On 15 May the U.S. House of Representatives passed the HEROES Act,^2^ but U.S. Senate Majority Leader McConnell refused to allow the Senate to consider it or a revision the House of Representatives passed on October 1. If the Senate had passed and the President signed it, the legislation would have enabled state and local health departments to undertake massive contact tracing of SARS-CoV-2, preventing many Covid-19 cases and deaths.

## Deregulation: emergency use authorization (EUA)

First established under President George W. Bush in 2004, the emergency use of diagnostics and therapeutics had its origin in the 1980s. Pressured by gay men who demanded the rapid availability of new drugs to treat HIV-AIDS, the FDA relaxed its stringent regulations amid bitter controversy. The 2004 law permitted the FDA to approve the use of new drugs and diagnostics without meeting its usual requirements in the event of a public health emergency.

The Trump Administration declared such an emergency for Covid-19 on 31 January 2020. On 4 February, FDA issued its first EUA to CDC for its reverse transcriptase-polymerase chain reaction (RT-PCR) test kit to detect the SARS-CoV-2 virus. Remarkably, the EUAs for RT-PCR waived FDA’s “good manufacturing practice requirements, including the quality system requirements … with respect to the design, manufacture, packaging, labeling, storage, and distribution of your product” [7]. The agency went even further at the end of March, announcing that it had “revised the [EUA] process to allow labs to begin testing *prior to FDA review* of their validation data. This policy change was an u*nprecedented* action to expand access to testing (*emphasis added)*” [8].

Under its EUA, CDC shipped PCR test kits to public health laboratories “before QC [quality control] had been conducted on them … (T)his might have compromised sufficient QC … to identify … the possibility of contamination before shipment” [9]. Of the 26 health department laboratories to whom CDC delivered RT-PCR kits in early February, 24 promptly discovered that the positive control vials in the kits were contaminated with SARS-CoV-2, yielding false positive test results [10]. By 10 February, CDC recalled the contaminated kits before they had been used to test patients. By 29 February, CDC and state and local health department laboratories were able to provide validated PCR tests.

By early March, FDA began to receive applications for EUAs for diagnostic tests from commercial laboratories and manufacturers, approving the first of them on 12 March. Other approvals quickly followed and by 15 December 2020 FDA had issued 199 EUAs for SARS-CoV-2 tests. Most EUAs went to commercial in-house testing laboratories and test-kit manufacturers, with a few to non-profit hospitals and research laboratories as well as to health department laboratories [11]. In previous recent epidemics in the U.S., most if not all diagnostic testing was done in public health department laboratories using CDC test kits.

The low bar to launching a test under an EUA made it easy for organizations with questionable expertise to enter the testing arena. In Utah, Nebraska, Iowa, and Tennessee, evidence of fraud by a testing consortium that had been awarded millions of dollars in state contracts quickly surfaced when it could not deliver the quantity of tests promised or attain the level of validity as tests in other laboratories [12, 13].

The ease with which EUAs for SARS-CoV-2 tests could be obtained also stimulated innovations in PCR tests and in antigen tests for the virus’s spike protein. However, at the time the first EUAs were issued, a valid PCR test was all that was needed to cope with the pandemic. By opening the floodgates to private investment, the EUAs not only led to corrupt practices and poorly validated tests, but stymied effective contact tracing by public health organizations.

Had the Trump administration given CDC more resources for quality-assured RT-PCR test production so that it and state and local health departments were capable of performing most diagnostic tests in the U.S. rather than opening the field to commercial laboratories and manufacturers, the pandemic could have been quickly contained. The Trump administration also showed its predilection for private industry when it demanded that CDC delete an app from its website that allowed Americans to screen themselves for symptoms of Covid-19. Instead, the administration worked with the Apple corporation to develop a similar tool [14].

FDA also issued EUAs for two drugs to treat Covid-19 patients. Political intrusion was clearly evident in the first, the anti-malarial hydroxychloroquine. FDA issued an EUA for it on 28 March and the Trump Administration promoted even broader distribution of the drug than the EUA authorized. Because of serious adverse events and lack of efficacy, FDA withdrew the EUA in June 2020 [15].

The EUA for the second drug, Remdesivir, ended on October 22, when FDA approved its use in Covid-19 patients, the first drug to receive that status. In contrast to FDA’s plan to convene an advisory committee before approving vaccines for SARS-CoV-2 (see below), the agency did not convene its Antimicrobial Drugs Advisory Committee before approving Remdesivir although several controlled clinical trials, including a very large one by the World Health Organization, found that at best the drug shortened the duration of mild illness by a few days. It did not consistently reduce the viral load or the chance of death from the infection [16]. Approval could bring its manufacturer, Gilead Sciences, more than $1 billion. Gilead’s price for a course of Remdesivir therapy for Covid-19 is $3120 [17].

On 11 November 2020, FDA granted an EUA to Eli Lilly Co. for a monoclonal antibody against a SARS-CoV-2 protein for treating mild to moderate covid-19 but warned that hospitalized Covid-19 patients or those requiring oxygen therapy to whom the drug was administered might have poorer outcomes [18].

## Diagnostic testing

With over 100 laboratories performing diagnostic tests in the U.S., and with the United States second only to China in the total number of tests, and tests per capita^3^ reported for SARS-CoV-2 [1], a shortage of effectively used tests seems an unlikely explanation for why the U.S. remained the country with the most Covid-19 cases and deaths.

The tests were not used effectively. *The Atlantic* stated the major reason for the U.S. failure to contain the pandemic:

Our reporting has unearthed a new coronavirus-testing crisis. Its main cause is not the federal government, nor state public-health labs, but *the private companies* that now dominate the country’s testing capacity. Testing backlogs have ballooned, slowing efficient patient care and delivering a heavily lagged view of the outbreak to decision makers (*emphasis added*) [19].

Without adequate public health resources, U.S. vulnerability to the virus has been at the mercy of the market.

Test inaccuracies are another factor contributing to the U.S. failure to contain its pandemic. Some false negative test results could have been avoided if FDA had not waived good manufacturing/QC practices. Two independent academic laboratories found that PCR tests for detecting SARS CoV-2 differed by as much as 15% in the frequency of false negatives [20]. EUA-authorized laboratories also differed in the amount of SARS-CoV-2 they could detect by over one-thousand fold [21]!

On 16 December FDA approved a home test for SARS-CoV-2 that does not require a doctor’s prescription. Although an app is required to get the result, the home test may result in undercounts of the number of infections and reduce the effectiveness of contact tracing. The test also costs about $30, putting it out of reach of people at great risk of infection [22].

## Contact tracing

Contact tracing^4^ and quarantine initially contained the epidemics in several countries, including China, South Korea, and New Zealand. Unfortunately, neither the CDC nor state health departments had sufficient funds to mount effective contact tracing for Covid-19. Meanwhile, commercial labs, which perform the majority of tests for SARS-CoV-2 in the U.S., have done little contact tracing. With the additional human resources needed it would be very costly and yield virtually no profit to them. Moreover, a recent survey found that “COVID-19 test result times have gotten faster but are still too slow to support broad contact tracing” [23]. The Trump White House scuttled plans to trace contacts of people present at a White House ceremony with President Trump at which he appeared healthy but was infected with the virus [24].

Contact tracing may not be as effective despite the resurgence of Covid-19 as the public has become more restive with business and school closures, and restrictions on travel and large gatherings. As the pandemic progressed, the 30% gap between the percentage of Americans who were concerned that businesses were opening too quickly (63%) rather than too slowly (33%) at the beginning of July shrank to 12% at the end of August [25]. Post-voting polls on November 3 showed still less of a gap [26].

## Inequality

Another reason the United States has fared so poorly is that the people at greatest risk of being infected by the virus have not been as able to protect themselves from infection as others. As of July 2020, Blacks and Hispanics had respectively 1.9 and 2.8 times the rate of positive SARS-CoV-2 tests as Whites. Blacks and Hispanics also had respectively 3.3 and 4.1 times as many hospitalizations per capita once they were diagnosed and both groups had 2.4 times as many deaths per capita as Whites [23].

The higher rates were not because these groups did not wear masks. Blacks and Hispanics reported higher use of cloth face masks within a week after CDC recommended them on 3 April: Blacks 74.4%, Hispanics 77.3%, compared to 54.3% using cloth masks among non-Hispanic Whites [27].

One reason for their higher rates is that many Black and Hispanic people live in overcrowded, low income neighborhoods where it is harder to limit contacts with others. In April, 38% of people living in neighborhoods in the lowest income quintile spent the entire day at home, compared to 47.1% living in the highest quintile. Conversely, 12.6% in the lowest quintile worked outside the home compared to 10.2% in the highest quintile [28].

Despite their greater chance of contracting Covid-19, Blacks and Hispanics received only 1.2 and 1.1 times as many SARS-CoV-2 tests respectively as whites. Test results took a day longer to be reported to Blacks than to Whites and only 56% of all those who tested positive reported being notified for contact tracing [23].

COVID-19 has disproportionately affected people who are incarcerated in the U.S. of whom African Americans and Hispanics comprise 56% but only 32% of the population [29]. At the beginning of June, the COVID-19 case rate in prisons was 5.5 times higher and the age-adjusted death rate 3 times higher than that of the overall U.S. population. The average daily increase in Covid-19 cases between 1 March 2020 and the beginning of June was 8.3% in prisons compared to 3.4% in the general U.S. population [30].

Federal laws require insurers to pay for the cost of testing for most of those with health insurance (including Medicare and Medicaid). For the uninsured in low and middle income groups, the federal government will reimburse for the test, but such users usually have to pay upfront at the time of testing, deterring many people from being tested. In one large study, test prices ranged between $20 and $850 per test, with a median of $127 [31]. Many testing sites across the country required people to be tested in their cars, making it difficult for people using mass transit or coming on foot to be tested. Nor does the federal government reimburse for ancillary services used with testing, such as personal protective coverings for health care workers administering the tests.

Blacks and Hispanics comprise 45% of essential workers [32] but, as already stated, only 32% of the population. In April 2020, the U.S. Department of Labor proposed to relieve employers of the obligation to record Covid-19 infections among workers. If this had gone into effect it would have been difficult to establish that the infections were work-related. Even without it, inspections by the Occupational Safety and Health Administration (OSHA) were cursory in industries declared essential. OSHA also reduced the number of inspectors during the emergency. In at least one instance, a meat-processing plant’s director was given a heads-up the day before an “un-formal” inspection. Eventually, two slaughterhouses were minimally fined, one for $13,494, the other for $15,615, as a result of 12 Covid-19 deaths and 2,000 workers with positive test results. As a former OSHA Chief of Staff told *The New Yorker,* “These are Black and brown workers. I just don’t think this Administration cares about them at all” [33].

While unemployment remains high, average hourly wages have fallen from April to October 2020 [34], but billionaires in the United States saw their net worth rise by almost $1 trillion in the first 9 months of the pandemic [35]. Between April and September 2020, 45 of the 50 most valuable publicly traded U.S. companies turned a profit; 27 of the largest 50 had layoffs. Several of the companies that initially pledged not to lay off workers during the pandemic ended up doing so. One of these companies, Salesforce, laid off 1000 workers in August 2020 while generating $2.7 billion in profit during the first 6 months of the pandemic, a 33% increase over its profits during the same period in 2019 [36].

## Vaccines

Unlike the situation near the start of the Covid-19 pandemic, when RT-PCR—the scientific basis for a valid diagnostic test for the SARS-CoV-2 virus—was available, no single path to a safe and effective vaccine against the virus was known. Thus it made sense for governments to encourage government and private research laboratories to each develop its own approach to manufacturing a vaccine. In May, the Department of Health and Human Services (DHHS) and the Department of Defense established Operation Warp Speed (OWS) with experts

in development, manufacturing, and distribution to work in close coordination. The initial set of ambitious objectives: to deliver tens of millions of doses of a SARS-CoV-2 vaccine — with demonstrated safety and efficacy, and approved or authorized by the FDA for use in the U.S. population — beginning at the end of 2020 and to have as many as 300 million doses of such vaccines available and deployed by mid-2021 …OWS leverages the full capacity of the U.S. government to ensure that no technical, logistic, or financial hurdles hinder vaccine development or deployment …OWS is supporting the companies financially and technically to commence process development and scale up manufacturing [37].

Instead of issuing EUAs to give companies free rein in vaccine development, as FDA had done for SARS-CoV-2 RT-PCR tests, OWS first established stringent criteria that pharmaceutical companies had to meet in conducting large scale randomized controlled clinical trials and have efficacy outcomes by early 2021. Only then could they apply to FDA for approval to provide their respective vaccines outside an experimental setting. OWS signed contracts (discussed below) with large pharmaceutical companies to develop and distribute their vaccines. Three companies reached phase 3 of their clinical trials over the summer of 2020 and three others were anticipated to begin phase 3 trials by the end of that year. OWS intentionally chose companies that based their vaccines on any one of four promising platform technologies. Two of the three companies farthest along in phase 3 trials (Pfizer and Moderna) used an mRNA platform while the third (AstraZeneca) used a replication-defective live-vector platform [37].

With unprecedented speed, OWS has achieved its goal for 2020. Pfizer [38] and Moderna [39] established that two doses of their respective mRNA vaccines injected a few weeks apart prevent over 90% of Covid-19 infections in the total population sampled and applied for EUAs for their vaccines. FDA’s guidelines for companies applying for EUAs for Covid-19 vaccines are much more stringent than the EUAs it issued for diagnostic tests, containing no waivers and no compromise with quality [40]. Each applicant must continue its clinical trial, and its EUA application must include information on the trial’s interim results on safety and efficacy, fact sheets for providers and vaccine recipients, and plans for distribution.

Applications for an EUA has been or will be reviewed by FDA’s Vaccines and Related Biological Products Advisory Committee (VRBPAC) before being approved. All voting members of VRBPAC are, by regulation, without industry affiliations [41].

As of this date, Pfizer and Moderna’s EUA applications have been reviewed by VRBPAC. In both cases, FDA asked the committee one question: Do the benefits of the company’s vaccine exceed the risks? Many media reports interpret the affirmative but not unanimous votes as recommending issuance of the respective EUAs. The VRBPAC’s recommendations undoubtedly helped FDA reach its decision—only occasionally does FDA make a decision against the advice of its advisory committees—but the agency must take factors other than the benefit:risk ratio into consideration.

Several factors could reduce the benefit of a vaccine in the real world compared to its efficacy in randomized controlled clinical trials: [42] (1) The ongoing clinical trials may still not have enough volunteers in minority groups, elderly people, and teenagers to have as much statistical confidence in the vaccines’ effectiveness in them as in the sample as a whole. (2) The vaccines may provide only partial immunity, preventing, for instance, more mild than serious cases and deaths, or immunity may not be long-lasting. (3) Not everyone eligible to receive the vaccine will agree to take it. In a recent poll, 59% of White but only 43% of Black Americans indicated they would get the vaccine when it is available [43]. (4) Not everyone will have access to the vaccine. Although “(a)ny vaccine … purchased with US taxpayer dollars will be given to the American people at no cost”, [44] distribution problems may delay or ultimately fail to reach all those at risk [45]. (5) Vaccine lots may become inactivated before they can be injected. This is most likely for Pfizer’s vaccine which requires storage at -70 degrees Celsius [38], making it impractical in some poor, rural areas [46].

The safety of anti-viral vaccines—as measured by the frequency of serious adverse events—will take longer to establish than efficacy. Already, however, two of the first vaccinees in the U.K. and one in Alaska have suffered anaphylactic reactions and a fourth suffered a typical moderate allergic reaction with eye puffiness and scratchy throat [47]. All were treated promptly and recovered. The patient who developed anaphylaxis in Alaska had no history of previous severe allergic reaction. The two reactions in Alaska were reported through established surveillance modules from the same hospital on the same day, raising questions of whether other hospitals administering the vaccine have failed to report them and whether the two allergic reactions in Alaska may not have been due to the vaccine but to the vaccinator (e.g., her/his perfume or aftershave) or to some material used in preparing the vaccinees for the injection. It is puzzling that no allergic reactions have been reported in the clinical trials.

Although adverse events are likely to occur within 2 months of completing vaccination, they may be too rare to have been observed in the fewer than 100,000 participants in the clinical trials at the time of the first EUA approvals in December. The occurrence of serious side effects, like Guillain-Barre syndrome in about 1 person in 1.5 million vaccinated for the H1N1 influenza virus in 2009 and about 1 in 100,000 in a 1976 H1N1 epidemic [48], could take hundreds of thousands of Covid-19 vaccinations before it would even be suspected and surveillance of millions of vaccinated people before it could be associated with the vaccine.

Although it is likely that the benefits of the Pfizer and Moderna vaccines, will continue to outweigh the risks, continued surveillance is needed. This will be hampered if volunteers in the ongoing blind clinical trials who received placebo rather than vaccine elect to get vaccinated, making it statistically difficult to compare the frequency of adverse reactions in the two groups.

As already noted, OWS has funded pharmaceutical companies to develop and deploy their vaccines. Moderna received almost $1 billion in April and AstraZeneca received $1.2 billion for development in May. Although Pfizer declined government support for development of its vaccine [38], DHHS announced in July that Pfizer will receive up to $1.95 billion for large-scale manufacturing and distribution of 100 million doses of its vaccine contingent on FDA approval. “The federal government will own the 100 million doses of vaccine initially produced as a result of this agreement” [44]. In August, DHHS announced $1.5 billion to support large-scale manufacturing and distribution of Moderna’s vaccine, stating that it will own the resulting 100 million doses of vaccine Moderna produces [44]. In December, DHHS signed an agreement with Moderna for an additional 100 million doses and bringing total support for Moderna’s distribution to $4.1 billion. DHHS has provided support for vaccine development and/or distribution with AstraZeneca, Johnson & Johnson ad Sanofi/GlaxoSmithKline [44].

Both Pfizer and Moderna “expect to profit, and not to provide their [vaccine] products at cost” [38]. Under the OWS contracts, they cannot charge for the hundreds of millions of vaccine doses they turn over to the federal government. Aside from profit Pfizer has made in its contract for distribution and Moderna has made in its contracts for development and distribution, these companies can profit from sales in other countries or in the U.S. after their contracts expire. In addition to the subsidies these companies obtained under OWS, the decades of underlying basic research in government laboratories, notably at NIH, has been paid for by U.S. taxpayers.

One sour note in Operation Warp Speed is a potential conflict of interest of its chief adviser, Moncef Slaoui. In his disclosure for the paper on OWS that he co-authored [35] he declared he was “formerly on the board of directors of Moderna, one of the companies with a selected vaccine for Covid19” [35]. The day before President Trump announced Slaoui’s appointment as OWS’s chief adviser, he resigned from Moderna’s board [49].

## The future

The United States and many other countries are into their third surge of Covid-19 cases, which in most countries, exceeds the previous two surges in number of new cases and deaths. Despite over 1 million SARS-CoV-2 lab tests currently being performed per day in the U.S. [50], the epidemic continues to increase exponentially [1].

China and New Zealand, two of the countries mentioned earlier that had curbed their pandemics, have not yet had a second surge [1]. Their continued successes can be attributed primarily to testing, contact tracing, and quarantine, all done under government initiative. In the U.S. most tests are performed in commercial laboratories that have little incentive for contact tracing. With a public increasingly impoverished by unemployment and impatient at being cooped up, the public may be less receptive to contact tracing and quarantine than would have been the case at the start of the pandemic. What, then, are the prospects for reversing the increase in Covid-19 cases in the U.S.?

The remarkable success of Operation Warp Speed in making two efficacious SARS-CoV-2 vaccines available to high risk groups in record time offers a glimmer of hope. What at first seems even more astounding is that President Trump’s government became not only sole owner of the vaccines but also the single payer for them, replacing health insurers and out-of-pocket payers. This is not so astounding when we realize that the recipient of this government largesse is the pharmaceutical industry whose rate of profit is higher than other industries [51].

For reasons already stated, the effectiveness of these vaccines and others may not be as great as expected from the phase 3 clinical trials. The hurdles already encountered in distributing the vaccines, plus the persistent suspicions many people have about the vaccines, will reduce the rate at which people become immune. In addition, some vaccine recipients may get a false sense of security about getting infected from the large unvaccinated portion of the population and will no longer wear masks or safe distance. We still don’t know whether the vaccine prevents vaccinees from acquiring asymptomatic infections that they can communicate to others who are not immune.

Over the past 9 months President Trump and his administration have issued contradictory information on how the pandemic can be curbed. Often they have been at odds with state governments, leaving the public confused about what to do. When President-elect Biden takes office on 20 January 2021, the messages from the federal and many state governments will be consistent, but until the Congress passes, and the president signs, legislation authorizing sufficient funding for massive contact tracing and quarantine, the coming winter surge on top of the autumn one will not be contained. Making matters worse, the number of people living in poverty, among whom people of color comprise a disproportionate share, rose by 8 million between May and October in the U.S. [52], and will continue to grow, increasing the number of people most vulnerable to Covid-19 infections.

Without Congressional action, the 13 million people receiving payments under programs enacted by Congress last spring will lose their remaining unemployment benefits when they expire at the end of 2020, as will a partial federal eviction moratorium [53]. As of 18 December, Congress has failed to agree on a relief package (although negotiations are ongoing) and aid to states to expand contact tracing. Unless both Democratic candidates for Senate from Georgia win in the runoff election on 5 January, President Biden will have a difficult time getting such legislation passed.

For the long term, negative and positive lessons can be learned from the Trump administration’s management of the Covid-19 crisis. Reducing the authority and capability of CDC and state and local health departments to test for SARS-CoV-2 and leaving it to commercial laboratories and manufacturers with only a modicum of federal regulation, was a disaster. On the other hand, the strong role federal government played in laying down stringent requirements for clinical trials that pharmaceutical companies had to meet before they could make vaccines available to the public, resulted in efficacious vaccines in record time. (Continued monitoring for adverse events is needed to assure safety.) These lessons will help future governments prepare for the inevitable occurrence of the next pandemic.

Menlo Park CA, USA

18 December 2020

## Data Availability

N/A

## Footnotes

1 Deaths per capita in countries with total populations over 1 million. As of 17 December 2020.

2 In addition to greatly increasing funds to health departments, the HEROES Act extended and supplemented unemployment payments and other benefits of the CARES Act to cope with the pandemic. The CARES Act was passed with bipartisan support by both houses of Congress and signed by President Trump in March 2020.

3 Tests per capita in countries with total populations over one hundred million. As of 17 December 2020.

4 Contact tracing entails systematically contacting infected individuals and interviewing them to identify others with whom they had been in contact while they were infectious. If diagnostic tests for the disease are available, the contacts are tested and quarantined for the duration of the incubation period, regardless of symptoms. (For Covid-19, asymptomatic contacts can be infectious.) Contacts of those whose test results are positive can in turn be tested.
